# High-sensitivity C-reactive protein and all-cause mortality in patients with diabetic foot and osteoporosis: evidence from a retrospective cohort study

**DOI:** 10.3389/fmed.2025.1700752

**Published:** 2026-01-12

**Authors:** Ting Liu, Gu-Gen Xu, Bo Chen, Ke-Jing Zeng, Yu Weng, Ping Li, Yi Xiao, Hai-Ming Liu, Mei-Qiu Fan, Peng-Cheng Chen

**Affiliations:** 1 Department of Endocrinology, The Affiliated Guangdong Second Provincial General Hospital of Jinan University, Guangzhou, China; 2 Department of Orthopedics, The Affiliated Guangdong Second Provincial General Hospital of Jinan University, Guangzhou, China

**Keywords:** all-cause mortality, diabetic foot, high-sensitivity C-reactive protein, inflammatory biomarkers, osteoporosis, risk prediction

## Abstract

**Objective:**

Diabetic foot (DF), often accompanied by osteoporosis, greatly increases the risk of disability and death. Inflammation is a key driver, with high-sensitivity C-reactive protein (Hs-CRP) serving as a marker of low-grade systemic inflammation. Yet, its prognostic role in this population remains unclear. This study aimed to evaluate the association between Hs-CRP and all-cause mortality in patients with osteoporosis complicated by DF.

**Methods:**

This investigation was a single-center retrospective cohort study that enrolled 468 hospitalized patients with osteoporosis complicated by DF at the Affiliated Guangdong Second Provincial General Hospital of Jinan University between July 2020 and August 2025. Cox proportional hazards models, subgroup and sensitivity analyses were employed to examine the independent association between Hs-CRP and all-cause mortality. In addition, receiver operating characteristic (ROC) curves and restricted cubic spline (RCS) modeling were applied to assess the predictive performance of Hs-CRP and to explore potential dose–response relationships.

**Results:**

During a median follow-up of 38.4 months, a total of 50 deaths were recorded. Cox proportional hazards regression demonstrated a significant positive association between Hs-CRP and all-cause mortality. After full adjustment for potential confounders, each 1 mg/L increase in Hs-CRP was associated with a 1.6% higher risk of death (HR = 1.016, 95% CI: 1.004–1.027, *p* = 0.008), while a one–standard deviation rise corresponded to a 30.7% increase in risk (HR = 1.307, 95% CI: 1.074–1.590, *p* = 0.008). Patients in the high Hs-CRP group exhibited nearly double the mortality risk compared with those in the low group. Subgroup analyses revealed that this association was particularly evident among individuals aged > 65 years, males, those with hypertension, or those without dyslipidemia. Sensitivity analysis excluding participants with impaired renal function yielded consistent results, supporting the robustness of the findings. The ROC curve indicated that Hs-CRP had moderate discriminatory ability for mortality prediction (AUC = 0.748, 95% CI: 0.686–0.811, *p* < 0.001). RCS analysis further confirmed a linear dose–response relationship between Hs-CRP and mortality risk (P-nonlinear = 0.910).

**Conclusion:**

Hs-CRP is independently associated with all-cause mortality in patients with osteoporosis complicated by DF, indicating its value for providing a potential tool for risk stratification.

## 1 Introduction

Diabetic foot (DF) is among the most severe complications of diabetes, characterized by infection, ulceration, and peripheral vascular disease, all of which profoundly worsen patient prognosis (1). Long-term follow-up studies have demonstrated that DF substantially increases the risk of all-cause mortality. For example, Al-Rubeaan and colleagues conducted a cohort-controlled study using data from the Saudi National Diabetes Registry, enrolling 840 diabetic foot ulcer (DFU) patients, and found that the all-cause mortality rate among DFU patients reached 18.5%, which was markedly higher than that observed in those without foot complications (2). Similarly, Rubio et al. followed 338 patients with newly diagnosed DFU for up to 12.2 years, reporting that 59.5% died with a five-year survival reduction of about 60%, and showing that ulcer severity was independent predictors of mortality (3). In addition, Vitale et al., drawing on the Renal Insufficiency And Cardiovascular Events (RIACE) cohort with 15,773 patients with type 2 diabetes mellitus (T2DM) followed for 7.42 years, reported that 5.7% had a history of DF and showed an approximately 50% higher risk of all-cause mortality, with the strongest association seen for amputation, followed by ulcer/gangrene, while isolated lower-limb revascularization carried a comparatively lower risk, and this relationship was independent of other complications (4). Beyond the direct risk imposed by DF, a growing body of evidence indicates that osteoporosis is also highly prevalent among patients with diabetes and particularly among those with DF. Studies have shown that individuals with diabetes frequently exhibit reduced bone mass and impaired bone quality, leading to a higher risk of osteoporosis and fragility fractures (5, 6). In DF patients, local bone demineralization, chronic inflammation, recurrent ulceration, and infection further exacerbate bone loss (7). International guidelines also emphasize that Charcot neuro-osteoarthropathy—characterized by inflammatory bone destruction, increased osteoclast activity, and joint collapse—significantly contributes to rapid localized osteoporosis in DF patients (8). Additionally, prolonged immobilization, abnormal weight-bearing due to neuropathy, and repeated microtrauma further promote bone resorption, making osteoporosis a common comorbidity in this population. These mechanistic and clinical observations collectively explain why DF is frequently accompanied by osteoporosis. Osteoporosis, in turn, raises the likelihood of fractures, disability, and mortality, thereby establishing a vicious cycle of “bone fragility–complications–death.”

Inflammation is a unifying mechanism that drives the progression of diabetes, osteoporosis, and DF. High-sensitivity C-reactive protein (Hs-CRP), a well-established biomarker of low-grade systemic inflammation, has been consistently associated with adverse outcomes in diabetic populations (9). For example, Lin et al., using data from 2,332 patients with T2DM in the Taiwan Biobank, conducted a Mendelian randomization study and demonstrated a significant causal link between Hs-CRP levels and diabetic nephropathy (DN): observational analyses indicated that higher Hs-CRP was associated with increased DN risk, while Mendelian randomization (MR) analysis with genetic risk scores as instrumental variables further revealed that each one-unit rise in genetically predicted log-transformed Hs-CRP markedly elevated DN risk, suggesting that elevated Hs-CRP may act as a causal risk factor for DN in patients with T2DM (10). In the specific context of DF, CRP has been widely used as a marker of infection severity. For example, Zakariah et al., in a study of 128 patients with DFU, observed that individuals with infected diabetic foot ulcers (IDFU) had markedly higher serum levels of Hs-CRP, which also showed the greatest diagnostic performance among all tested markers, with an area under the curve (AUC) of 0.91; the optimal cut-off value was 3.47 mg/dL, providing 80% sensitivity and 89% specificity for identifying infection, indicating that Hs-CRP is a more sensitive diagnostic indicator than procalcitonin in detecting infection in DFU (11). Despite growing evidence that Hs-CRP is associated with diabetes outcomes, DF severity, and fracture risk, no study to date has systematically investigated whether Hs-CRP independently predicts all-cause mortality in patients with the dual burden of osteoporosis and DF. This evidence gap is clinically relevant because conventional risk factors—such as age, and renal function—may be insufficient to fully capture the risk profile in this complex population. If Hs-CRP could serve as an independent predictor of mortality in such patients, it would open new perspectives for risk stratification, monitoring, and possibly inflammation-targeted interventions.

Therefore, the present study aims to examine the relationship between baseline Hs-CRP levels and all-cause mortality in hospitalized patients with DF complicated by osteoporosis, and to clearly define this association as the primary objective of the study, with the ultimate goal of providing evidence to support more accurate prognostic assessment and individualized management in this high-risk group.

## 2 Methods

### 2.1 Study population

This retrospective cohort study included 468 consecutive patients admitted between July 2020 and August 2025 at The Affiliated Guangdong Second Provincial General Hospital of Jinan University. As this study was based on a real-world retrospective design, all eligible patients during the study period were consecutively included. Therefore, the sample size was determined by the number of available cases rather than by *a priori* sample size calculation. This approach ensured that the cohort reflected the actual clinical population and captured all patients meeting the predefined criteria. Patients were eligible if they met the following criteria: (1) age > 40 years; (2) diagnosis of DF according to the International Working Group on the Diabetic Foot (IWGDF) criteria (12); and (3) osteoporosis with a clearly established diagnosis in accordance with the National Bone Health Alliance (NBHA) Working Group position statement (13). Exclusion criteria were as follows: (1) acute or chronic inflammatory diseases unrelated to diabetes that could affect Hs-CRP levels; (2) active malignancy or ongoing chemotherapy; (3) severe hepatic failure or advanced non-diabetic renal disease (such as uremia); (4) severe hematological disorders (e.g., leukemia, aplastic anemia) or immune system diseases (e.g., systemic lupus erythematosus, rheumatoid arthritis); and (5) incomplete baseline data (such as Hs-CRP) or loss to follow-up. The study protocol was approved by the institutional ethics committee of The Affiliated Guangdong Second Provincial General Hospital of Jinan University, and written informed consent was obtained from all participants in accordance with the Declaration of Helsinki.

### 2.2 Measurement, assessment, and grouping of Hs-CRP

Fasting venous blood samples were obtained at admission, and serum Hs-CRP concentrations were determined in the hospital’s central laboratory using an immunoturbidimetric assay with a detection limit of 0.1 mg/L; intra- and inter-assay coefficients of variation were < 5%. For analytical purposes, Hs-CRP was assessed both as a continuous variable and in categorical groupings. To account for skewed distribution, log_10_-transformed values and standardized scores were also applied.

For categorical analyses, two grouping strategies were employed. First, patients were divided by the cohort-specific median Hs-CRP level (1.43 mg/L), resulting in a low Hs-CRP group (≤ 1.43 mg/L, *n* = 234) and a high Hs-CRP group (> 1.43 mg/L, *n* = 234). The median value was used primarily to ensure balanced sample sizes between groups, facilitating stable comparisons in baseline and subgroup analyses. Second, the optimal prognostic cut-off was determined using receiver operating characteristic (ROC) curve analysis, which identified 1.63 mg/L as the most discriminatory threshold; based on this value, patients were classified into a low Hs-CRP group (≤ 1.63 mg/L, *n* = 247) and a high Hs-CRP group (> 1.63 mg/L, *n* = 221). This threshold was chosen because it yielded the highest Youden index in ROC analysis, indicating the best overall discrimination for mortality prediction.

### 2.3 Definition and ascertainment of all-cause mortality

The primary endpoint of this study was all-cause mortality, defined as death from any cause during the follow-up period, irrespective of its underlying etiology. Mortality information was obtained from hospital electronic medical records, supplemented by outpatient follow-up visits and telephone interviews with patients or their relatives. To ensure full transparency in line with STROBE recommendations, we further clarified the follow-up procedures: follow-up commenced at the date of discharge from the index hospitalization, and all participants were systematically contacted at regular intervals through outpatient visits or structured telephone follow-up. Follow-up commenced at the date of discharge from the index hospitalization and continued until death or the end of the study period (August 2025), whichever came first. The overall follow-up duration and completeness were documented, and loss-to-follow-up cases were recorded and handled according to predefined procedures.

### 2.4 Baseline clinical and laboratory characteristics

Baseline demographic information, lifestyle factors, comorbid conditions, anthropometric indices, and laboratory parameters were systematically collected at the time of admission. Smoking status was defined as current or former habitual tobacco use. Comorbidities were diagnosed according to established clinical criteria. Hypertension was defined as a documented history of hypertension, current use of antihypertensive medication, systolic blood pressure (SBP) ≥ 140 mmHg, or diastolic blood pressure (DBP) ≥ 90 mmHg (14). Dyslipidemia was defined as a previous clinical diagnosis, current use of lipid-lowering therapy, or abnormal lipid profile according to guideline-recommended thresholds (triglycerides ≥ 2.3 mmol/L, total cholesterol ≥ 6.2 mmol/L, low-density lipoprotein cholesterol [LDL-C] ≥ 4.1 mmol/L, or high-density lipoprotein cholesterol [HDL-C] < 1.0 mmol/L) (15). Use of antihypertensive or lipid-lowering drugs was also documented.

Anthropometric data included body mass index (BMI), calculated as weight (kg) divided by the square of height (m^2^). Blood pressure was measured on admission using a calibrated sphygmomanometer, and both SBP and DBP were recorded. Hematologic indices included white blood cell (WBC) count, neutrophil count, lymphocyte count, monocyte count, hemoglobin concentration, and platelet count. Biochemical measurements comprised fasting blood glucose (FBG), glycated hemoglobin (HbA1c), triglycerides, total cholesterol, LDL-C, HDL-C, uric acid, estimated glomerular filtration rate (eGFR), alanine aminotransferase (ALT), aspartate aminotransferase (AST), total bilirubin, serum albumin, and fibrinogen. These parameters were measured in the hospital’s central laboratory using standardized methods.

### 2.5 Statistical approach

Data processing and analysis were conducted with R software (version 4.4.3; R Foundation for Statistical Computing, Vienna, Austria) and SPSS Statistics (version 28.0; IBM Corp., Armonk, NY, United States). Continuous variables conforming to normal distribution were expressed as mean ± standard deviation, whereas those not normally distributed were reported as median and interquartile range. Categorical data were summarized as counts and percentages. Comparisons between survival and non-survival groups were performed using the independent-samples t test or the Mann–Whitney U test for continuous variables, and chi-square or Fisher’s exact tests for categorical variables.

Associations between Hs-CRP and mortality were examined using Cox proportional hazards regression. Hs-CRP was incorporated into the models as a raw continuous measure, as a log_10_-transformed value, and as a standardized variable per one standard deviation (SD) increment. In addition, categorical analyses were carried out using both the cohort-specific median cut-off (1.43 mg/L) and the threshold determined by ROC curve analysis (1.63 mg/L). Three progressive adjustment models were established. Model 1 included age; Model 2 further accounted for hypertension; and Model 3 additionally adjusted for lymphocyte count, hemoglobin, FBG, HbA1c, HDL-C, uric acid, eGFR, albumin, and fibrinogen. To ensure the validity of the Cox proportional hazards regression model, the proportional hazards assumption was formally evaluated using Schoenfeld residual–based tests. A *p* value greater than 0.05 was considered indicative of no violation of the proportional hazards assumption. Subgroup evaluations stratified by age, sex, hypertension, and dyslipidemia were undertaken with the same adjustment strategy. To verify the robustness of the findings, a sensitivity analysis was performed after excluding patients with renal insufficiency (eGFR < 60 mL/min/1.73 m^2^). Predictive performance of Hs-CRP for mortality was assessed by plotting ROC curves and calculating the AUC. Furthermore, restricted cubic spline (RCS) regression with three knots was used to visualize and test for potential dose–response associations. Results were presented as hazard ratios (HRs) with corresponding 95% confidence intervals (CIs), and a two-sided *p* value < 0.05 was considered statistically significant.

## 3 Results

### 3.1 Baseline characteristics by mortality status

Among the 468 patients with osteoporosis complicated by DF, a total of 50 deaths from all causes occurred during follow-up (Table 1). Patients who died were older than survivors (*p* < 0.001). The prevalence of smoking and hypertension were higher in the mortality group (*p* < 0.05). With respect to laboratory findings, individuals who died had lower lymphocyte counts, hemoglobin and albumin (*p* < 0.05), whereas plasma fibrinogen levels were elevated (*p* = 0.005). Markers of metabolic control and renal function were also less favorable in the mortality group, including higher HbA1c, increased uric acid, and reduced eGFR. Regarding liver function, ALT levels were lower among deceased patients (*p* = 0.042). In terms of lipid profile, HDL-C was significantly reduced (*p* = 0.025). Most notably, Hs-CRP levels were markedly higher in the mortality group (*p* = 0.001). By contrast, no significant differences were observed between the two groups with respect to sex distribution, triglycerides, total cholesterol, LDL-C, or other baseline variables (*p* > 0.05).

**Table 1:** Baseline characteristics grouped by all-cause mortality in patients with osteoporosis complicated by diabetic foot.

Variables	All patients	Non-all-cause mortality	All-cause mortality	*p* value
N	468	418	50	
Age, years	66.07 ± 8.09	65.51 ± 7.71	70.78 ± 9.62	< 0.001
Sex, n (%)				0.314
Male	256 (54.7)	232 (55.5)	24 (48.0)	
Female	212 (45.3)	186 (44.5)	26 (52.0)	
Smoking, n (%)	121 (25.9)	7 (14.0)	114 (27.3)	0.043
Hypertension, n (%)	268 (57.3)	231 (55.3)	37 (74.0)	0.001
Dyslipidemia, n (%)	226 (48.3)	202 (48.3)	24 (48.0)	0.965
Antihypertensive drugs, n (%)	243 (51.9)	213 (51.0)	30 (60.0)	0.226
Lipid-lowering drugs, n (%)	89 (19.0)	77 (18.4)	12 (24.0)	0.342
BMI, kg/m^2^	25.45 (23.54, 27.53)	25.39 (23.44, 27.55)	25.99 (24.31, 27.47)	0.125
SBP, mmHg	135.00 (122.00, 150.00)	134.00 (121.00, 150.00)	141.00 (127.25, 154.50)	0.101
DBP, mmHg	76.00 (69.00, 83.00)	75.50 (69.00, 83.00)	77.00 (71.00, 81.00)	0.753
WBC, x10^9^/L	7.44 (6.12, 9.33)	7.47 (6.11, 9.33)	6.99 (6.34, 9.16)	0.705
Neutrophil count, x10^9^/L	4.63 (3.60, 6.39)	4.65 (3.57, 6.40)	4.63 (3.95, 6.47)	0.464
Lymphocyte count, x10^9^/L	1.86 (1.41, 2.35)	1.88 (1.46, 2.39)	1.56 (1.14, 2.12)	0.010
Monocyte count, x10^9^/L	0.51 (0.41, 0.64)	0.51 (0.41, 0.64)	0.57 (0.44, 0.72)	0.147
Hemoglobin, g/L	137.00 (126.00, 147.00)	138.00 (127.00, 148.00)	127.50 (108.75, 141.00)	< 0.001
Platelet count, x10^9^/L	218.50 (183.00, 251.00)	220.00 (183.75, 251.00)	205.50 (174.00, 244.25)	0.433
FBG, mmol/L	7.46 (6.21, 9.72)	7.45 (6.20, 9.60)	8.09 (6.53, 10.50)	0.117
HbA1c, %	7.70 (6.90, 8.90)	7.70 (6.90, 8.80)	8.40 (7.05, 9.33)	0.025
Triglycerides, mmol/L	1.46 (1.07, 2.04)	1.47 (1.07, 2.05)	1.42 (1.16, 1.77)	0.488
Total cholesterol, mmol/L	4.46 (3.71, 5.21)	4.47 (3.73, 5.22)	4.01 (3.54, 4.82)	0.172
LDL-C, mmol/L	2.53 (1.96, 3.18)	2.54 (1.98, 3.18)	2.45 (1.88, 3.25)	0.561
HDL-C, mmol/L	1.15 (0.98, 1.36)	1.16 (0.98, 1.38)	1.08 (0.92, 1.21)	0.025
Uric acid, μmol/L	321.10 (267.93, 386.00)	319.90 (267.00, 381.65)	342.15 (282.75, 414.93)	0.036
eGFR, mL/mim/1.73m^2^	81.44 (66.32, 108.09)	82.66 (69.11, 110.77)	67.00 (52.94, 87.64)	< 0.001
ALT, U/L	21.00 (15.00, 32.33)	21.60 (15.08, 32.68)	18.10 (12.00, 31.80)	0.042
AST, U/L	21.00 (16.00, 39.00)	21.00 (16.00, 39.00)	22.70 (15.53, 34.45)	0.802
Total bilirubin, μmol/L	14.00 (10.40, 18.39)	14.15 (10.50, 18.48)	12.84 (9.28, 16.76)	0.255
Albumin, g/L	39.10 (36.25, 42.38)	39.32 (36.70, 42.62)	36.51 (33.16, 40.16)	< 0.001
Fibrinogen, g/L	3.27 (2.76, 3.81)	3.25 (2.74, 3.78)	3.62 (2.93, 4.54)	0.005
Hs-CRP, mg/L	1.43 (0.50, 5.20)	1.24 (0.50, 4.82)	3.05 (0.97, 18.08)	0.001

BMI, body mass index; SBP, systolic blood pressure; DBP, diastolic blood pressure; WBC, white blood cell count; FBG, fasting blood glucose; HbA1c, glycated hemoglobin; LDL-C, low-density lipoprotein cholesterol; HDL-C, high-density lipoprotein cholesterol; eGFR, estimated glomerular filtration rate; ALT, alanine aminotransferase; AST, aspartate aminotransferase; Hs-CRP, High-sensitivity C-reactive protein.

### 3.2 Association between Hs-CRP and all-cause mortality

The proportional hazards assumption was evaluated using Schoenfeld residuals. As shown in Supplementary Table S1, none of the covariates violated the proportional hazards assumption, with all *p* values being greater than 0.05. The global test also indicated no evidence of violation (χ^2^ = 10.4, *p* = 0.581). These findings support the suitability of the Cox proportional hazards model for the present analysis. Cox proportional hazards analyses demonstrated a consistent positive association between Hs-CRP and all-cause mortality across all three models (Table 2). In Models 1 and 2, which were adjusted for age alone and for age plus hypertension, respectively, higher Hs-CRP levels were already significantly associated with increased mortality risk, regardless of whether Hs-CRP was modeled as a continuous, log-transformed, or standardized variable. In the fully adjusted Model 3, this association remained robust. Each 1 mg/L increase in Hs-CRP was associated with a 1.6% higher risk of all-cause mortality (HR = 1.016, 95% CI: 1.004–1.027, *p* = 0.008). Similar results were observed for log-transformed Hs-CRP (HR = 1.787, 95% CI: 1.168–2.735, *p* = 0.007) and standardized Hs-CRP (HR = 1.307, 95% CI: 1.074–1.590, *p* = 0.008).

**Table 2:** Association between Hs-CRP and all-cause mortality in patients with osteoporosis complicated by diabetic foot.

Variables	Model 1			Model 2			Model 3		
	HR	95% CI	*p* value	HR	95% CI	*p* value	HR	95% CI	*p* value
Hs-CRP	1.019	1.009–1.029	< 0.001	1.021	1.011–1.031	< 0.001	1.016	1.004–1.027	0.008
Log_10_Hs-CRP	1.988	1.341–2.947	0.001	2.106	1.411–3.144	< 0.001	1.787	1.168–2.735	0.007
Standardized Hs-CRP	1.391	1.176–1.644	< 0.001	1.442	1.217–1.709	< 0.001	1.307	1.074–1.590	0.008
Median-based grouping (1.43 mg/L)									
Low Hs-CRP (≤ 1.43 mg/L)	Reference			Reference			Reference		
High Hs-CRP (> 1.43 mg/L)	2.270	1.236–4.168	0.008	2.323	1.264–4.272	0.007	2.031	1.077–3.829	0.029
Optimal cut-off grouping (1.63 mg/L)									
Low Hs-CRP (≤ 1.63 mg/L)	Reference			Reference			Reference		
High Hs-CRP (> 1.63 mg/L)	2.274	1.251–4.135	0.007	2.326	1.278–4.236	0.006	2.013	1.076–3.766	0.029

Model 1 was adjusted for age only. Model 2 was adjusted for age and hypertension. Model 3 was adjusted for age, hypertension, lymphocyte count, hemoglobin, fasting blood glucose, glycated hemoglobin, high-density lipoprotein cholesterol, uric acid, estimated glomerular filtration rate, albumin, and fibrinogen. Hs-CRP, high-sensitivity C-reactive protein; HR, hazard ratio; CI, confidence interval.

Categorical analyses further confirmed the association between elevated Hs-CRP and increased mortality risk. Patients with Hs-CRP above the median (1.43 mg/L) consistently exhibited over a twofold higher mortality risk across all models, including in the fully adjusted Model 3 (HR = 2.031, *p* = 0.029). Comparable results were observed using the ROC-derived cutoff (1.63 mg/L), with the high Hs-CRP group again showing nearly double the risk (HR = 2.013, *p* = 0.029).

### 3.3 Subgroup analyses of the association between Hs-CRP and mortality

Subgroup analyses are summarized in Table 3. Among patients aged > 65 years, Hs-CRP was significantly associated with all-cause mortality (e.g., per 1 mg/L increase: HR = 1.017, *p* = 0.018; log-transformed: HR = 2.429, *p* < 0.001; per 1 SD: HR = 1.331, *p* = 0.018). However, no significant associations were observed in those aged ≤ 65 years. In sex-stratified analyses, significant associations were found in men (e.g., per 1 mg/L: HR = 1.024, *p* = 0.006; per 1 SD: HR = 1.511, *p* = 0.006), while results in women were non-significant. A similar pattern was observed for hypertension status, with clear associations in hypertensive patients but null findings among those without hypertension. Likewise, significant associations were present in participants without dyslipidemia (e.g., per 1 mg/L: HR = 1.027, *p* = 0.002; per 1 SD: HR = 1.589, *p* = 0.002), but not in those with dyslipidemia. It is important to note that several of the subgroups with non-significant findings—such as younger participants, females, or those without hypertension—had relatively few deaths, which may have limited statistical power and obscured true associations.

**Table 3:** Multivariate subgroup association between Hs-CRP and all-cause mortality in patients with osteoporosis complicated by diabetic foot.

Subgroups	N (Events)	Hs-CRP		Log_10_Hs-CRP		Standardized Hs-CRP		P for interaction
		HR (95% CI)	*p* value	HR (95% CI)	*p* value	HR (95% CI)	*p* value	
Age								0.906
≤ 65 years	220 (14)	1.005 (0.969–1.041)	0.804	0.734 (0.240–2.250)	0.589	1.081 (0.583–2.007)	0.804	
> 65 years	248 (36)	1.017 (1.003–1.031)	0.018	2.429 (1.525–3.869)	< 0.001	1.331 (1.051–1.686)	0.018	
Sex								0.381
Male	256 (24)	1.024 (1.007–1.042)	0.006	1.868 (0.948–3.681)	0.071	1.511 (1.123–2.034)	0.006	
Female	212 (26)	1.008 (0.987–1.030)	0.438	0.871 (0.389–1.949)	0.737	1.156 (0.802–1.667)	0.438	
Hypertension								0.771
Yes	268 (37)	1.020 (1.007–1.032)	0.002	1.716 (1.041–2.827)	0.034	1.400 (1.130–1.735)	0.002	
No	200 (13)	1.005 (0.978–1.032)	0.741	1.298 (0.434–3.883)	0.641	1.082 (0.679–1.724)	0.741	
Dyslipidemia								0.236
Yes	226 (24)	1.005 (0.986–1.025)	0.590	1.455 (0.678–3.123)	0.336	1.095 (0.788–1.521)	0.590	
No	242 (26)	1.027 (1.010–1.044)	0.002	1.536 (0.701–3.363)	0.283	1.589 (1.189–2.123)	0.002	

Subgroup analysis adjusted for age, hypertension, lymphocyte count, hemoglobin, fasting blood glucose, glycated hemoglobin, high-density lipoprotein cholesterol, uric acid, estimated glomerular filtration rate, albumin, and fibrinogen. Hs-CRP, high-sensitivity C-reactive protein; HR, hazard ratio; CI, confidence interval.

### 3.4 Sensitivity analysis after excluding patients with impaired renal function

To minimize the potential confounding effect of renal impairment, a sensitivity analysis was conducted after excluding patients with an eGFR < 60 mL/min/1.73m^2^ (Table 4). The findings indicated that the positive association between Hs-CRP and all-cause mortality remained robust. Specifically, each 1 mg/L increment in Hs-CRP corresponded to a 1.7% increase in mortality risk (HR = 1.017, *p* = 0.020). Similarly, the log-transformed Hs-CRP (Log_10_Hs-CRP) was significantly associated with mortality (HR = 1.868, *p* = 0.020), and each 1-SD increase in Hs-CRP was linked to a 33.2% higher risk of death (HR = 1.332, *p* = 0.020).

**Table 4:** Association between Hs-CRP and all-cause mortality in patients with osteoporosis complicated by diabetic foot: excluding patients with eGFR < 60 mL/min/1.73m^2.^

Variables	HR	95% CI	*p* value
Hs-CRP	1.017	1.003–1.031	0.020
Log_10_Hs-CRP	1.868	1.105–3.158	0.020
Standardized Hs-CRP	1.332	1.046–1.697	0.020
Median-based grouping (1.43 mg/L)			
Low Hs-CRP (≤ 1.43 mg/L)	Reference		
High Hs-CRP (> 1.43 mg/L)	2.168	1.011–4.648	0.047
Optimal cut-off grouping (1.63 mg/L)			
Low Hs-CRP (≤ 1.63 mg/L)	Reference		
High Hs-CRP (> 1.63 mg/L)	2.090	0.990–4.411	0.053

The sensitivity analysis was adjusted for age, hypertension, lymphocyte count, hemoglobin, fasting blood glucose, glycated hemoglobin, high-density lipoprotein cholesterol, uric acid, estimated glomerular filtration rate, albumin, and fibrinogen. Hs-CRP, high-sensitivity C-reactive protein; HR, hazard ratio; CI, confidence interval.

Analyses based on categorical variables yielded consistent results. When stratified by the median threshold of 1.43 mg/L, patients with Hs-CRP levels above this cutoff had approximately 2.17-fold higher mortality risk compared with those at or below the median (HR = 2.168, *p* = 0.047). Using the ROC-derived optimal cutoff of 1.63 mg/L, high Hs-CRP levels were likewise associated with an elevated mortality risk (HR = 2.090), though the association reached only borderline significance (*p* = 0.053).

### 3.5 Predictive value of Hs-CRP for all-cause mortality and dose–response association

**Figure 1:**
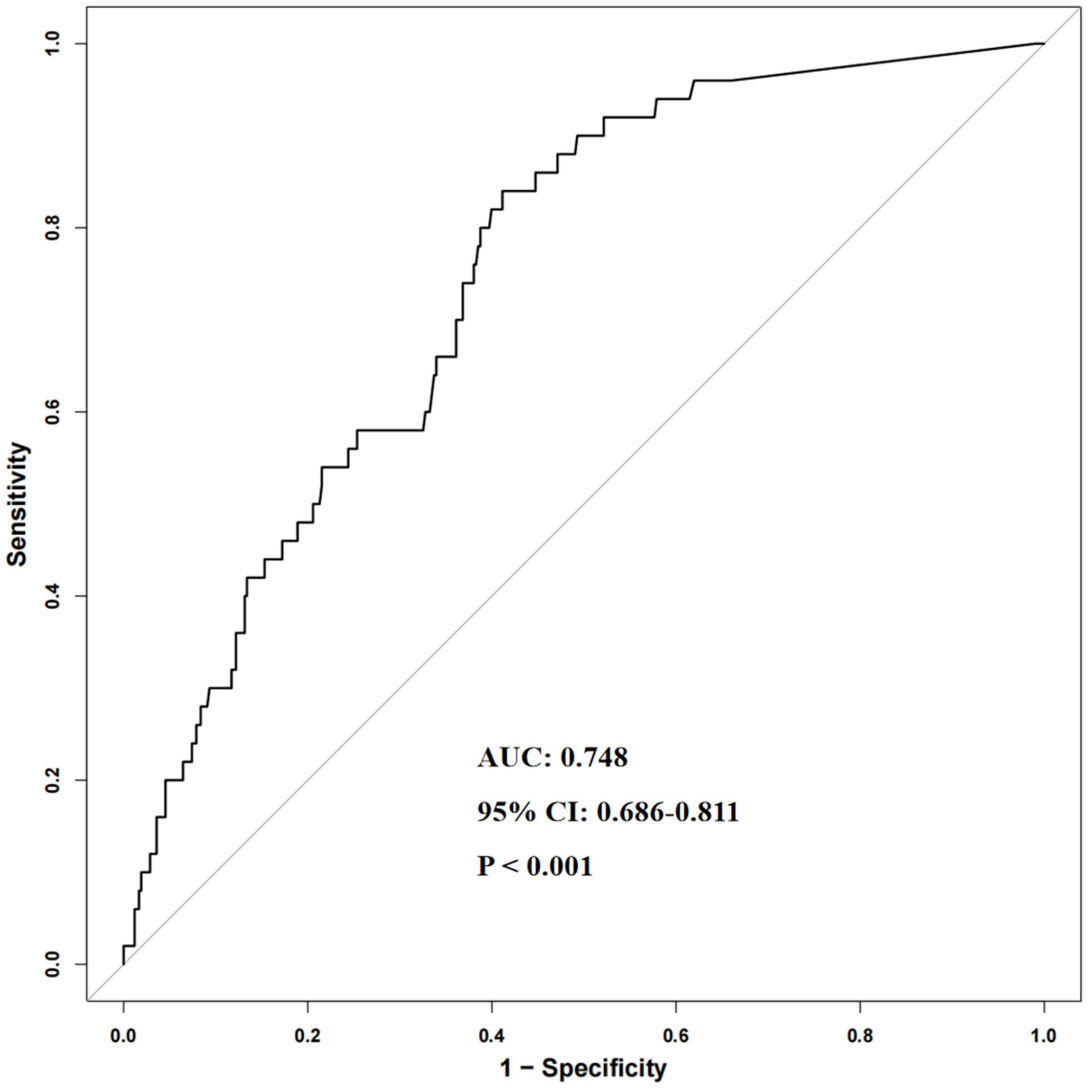
ROC curve showing the predictive value of Hs-CRP for all-cause mortality in patients with osteoporosis complicated by diabetic foot. ROC, receiver operating characteristic; Hs-CRP, high-sensitivity C-reactive protein; AUC, area under the curve; CI, confidence interval.

Figure 1 illustrated the ROC curve assessing the predictive performance of Hs-CRP for all-cause mortality in patients with osteoporosis complicated by DF. The findings indicated a moderate discriminative capacity, with an AUC of 0.748 (95% CI: 0.686–0.811, *p* < 0.001).

**Figure 2:**
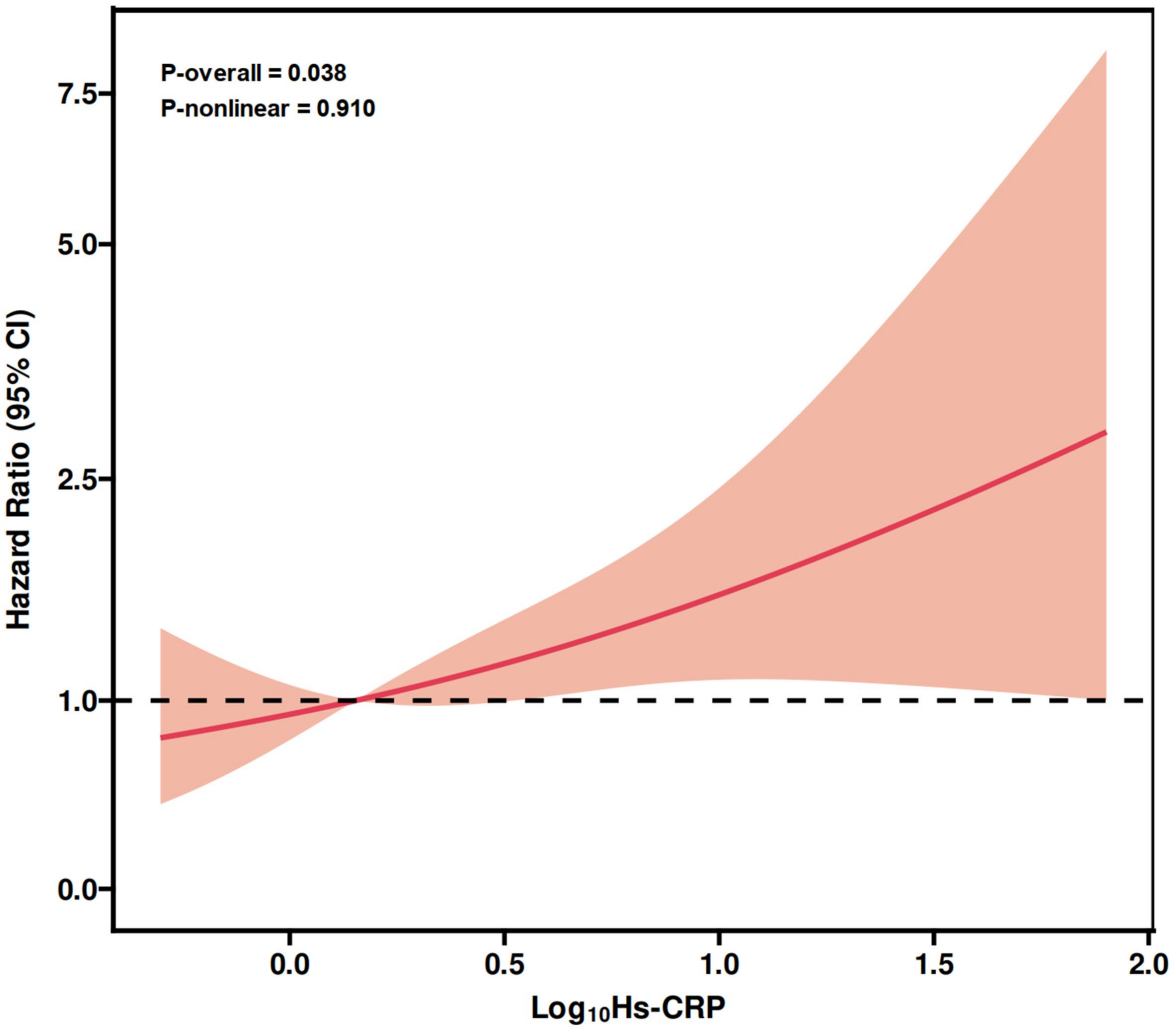
Restricted cubic spline plot showing the linear association between Hs-CRP and all-cause mortality risk in patients with osteoporosis complicated by diabetic foot. RCS, restricted cubic spline; Hs-CRP, high-sensitivity C-reactive protein; CI, confidence interval.

Figure 2 presented the RCS analysis, which demonstrated a linear association between Hs-CRP levels and mortality risk (P-overall = 0.038, P-nonlinear = 0.910). As Log_10_Hs-CRP increased, the risk of all-cause mortality showed a progressive upward trend.

## 4 Discussion

This study, based on a large cohort of 468 patients with osteoporosis complicated by DF, systematically evaluated the association between Hs-CRP and the risk of all-cause mortality. The findings demonstrated that elevated Hs-CRP levels were significantly associated with higher mortality risk, and this relationship remained robust even after comprehensive adjustment for multiple confounding factors. Subgroup and sensitivity analyses further reinforced these results. ROC curve analysis indicated that Hs-CRP had a moderate discriminatory ability in predicting patient outcomes, while RCS analysis confirmed a linear dose–response relationship between Hs-CRP and mortality risk. Collectively, these observations suggest that Hs-CRP represents a reliable, accessible, and clinically practical inflammatory biomarker for risk stratification and prognostic assessment. In patients with the dual burden of osteoporosis and DF—where conventional risk factors often fail to fully capture high-risk individuals—the integration of Hs-CRP into prognostic models may enhance clinical decision-making and management strategies. Overall, this study provides compelling evidence supporting the prognostic utility of Hs-CRP in this complex patient population, underscoring its potential clinical value.

Previous research has largely concentrated on the severity of infection in DF or on fracture risk in isolated osteoporosis, while evidence regarding the role of Hs-CRP in predicting long-term mortality among patients with both conditions remains lacking. Although prior studies have demonstrated strong associations between Hs-CRP and diabetes-related complications such as nephropathy and cardiovascular events (16, 17), systematic evidence supporting its prognostic significance in patients with osteoporosis complicated by DF has not been established. In recent years, only a limited number of studies have investigated the clinical utility of Hs-CRP in DF populations. For instance, Zhang and colleagues conducted a systematic review and meta-analysis of 25 studies and reported that the amputation rate among Chinese patients with DFU reached 22.4% (18). Subgroup analyses further revealed that inflammatory markers, including Hs-CRP, were significantly higher in amputated cases than in non-amputated cases, underscoring the role of inflammation in adverse DFU outcomes and suggesting a close link between elevated Hs-CRP and higher amputation risk. Similarly, Zakariah et al. studied 128 DFU patients and compared several infection-related biomarkers for their diagnostic value in identifying IDFU (11). They found that Hs-CRP had the highest diagnostic performance, with an AUC of 0.91 and an optimal cut-off of 3.47 mg/dL, yielding 80% sensitivity and 89% specificity—superior to both procalcitonin and WBC count—indicating that Hs-CRP has greater clinical value for infection detection. In another study, Todorova et al. included 119 diabetic patients (41 with IDFU, 35 with non-IDFU, and 43 with diabetes alone) and also demonstrated that Hs-CRP significantly outperformed procalcitonin in distinguishing infected from non-IDFU (19). This superiority was particularly evident in moderate infections and Wagner grade 3 ulcers, with AUC values of 0.856 and 0.911, respectively, whereas procalcitonin showed no significant differences across groups. Taken together, these findings suggest that Hs-CRP is closely associated with infection severity, amputation risk, and adverse short-term outcomes in DFU patients, and appears to outperform procalcitonin in diagnostic accuracy. However, existing evidence has been primarily limited to infection-related or amputation-related outcomes. Whether Hs-CRP can serve as an independent predictor of long-term survival in DFU patients, especially those with concurrent osteoporosis who represent a particularly high-risk subgroup, remains poorly understood. This gap highlights the need for well-designed prospective cohort studies and mechanistic investigations to further elucidate its prognostic value.

Unlike previous studies, this work offers several novel contributions. It is the first large-scale inpatient cohort to establish Hs-CRP as an independent predictor of all-cause mortality in patients with DF complicated by osteoporosis. The study employed multiple analytic strategies—including continuous, log-transformed, and standardized Hs-CRP measures—as well as two stratification methods (median-based and ROC-derived thresholds), all of which yielded consistent results, supporting the robustness of the findings. Subgroup analyses further highlighted stronger associations in older adults, males, hypertensive patients, and those without dyslipidemia. Moreover, a clear linear dose–response relationship between Hs-CRP and mortality was demonstrated. Together, these findings strengthen the evidence base for Hs-CRP as a feasible marker for individualized risk stratification and may inform future clinical and translational research targeting this high-risk population.

Hs-CRP, as a biomarker of systemic inflammation, reflects a state of chronic low-grade inflammatory activation and its association with mortality risk may be mediated through multiple biological pathways. First, patients with DF often suffer from persistent ulcers and recurrent infections, while coexisting osteoporosis further exacerbates local bone destruction (20). The overlap of these conditions leads to sustained inflammatory activation, with elevated Hs-CRP serving as a direct indicator of this process (21). Second, Hs-CRP can activate the complement cascade, impair endothelial function, and accelerate atherosclerotic plaque formation, thereby heightening the risk of cardiovascular and cerebrovascular events and ultimately contributing to increased all-cause mortality (22–24). Third, inflammatory signaling suppresses osteoblast activity while enhancing osteoclast-mediated bone resorption, which accelerates osteoporosis progression, elevates fracture and disability rates, and indirectly raises mortality risk (25). In addition, inflammation is closely linked to metabolic derangements such as insulin resistance, renal dysfunction, and hypoalbuminemia, all of which are key determinants of poor prognosis in patients with DF (26–31). Taken together, Hs-CRP is not merely a marker of inflammation but may actively participate in the cascade of multi-organ injury driven by the coexistence of DF and osteoporosis, lending biological plausibility to its role as an independent prognostic factor.

Despite the valuable insights provided by this study, several limitations should be acknowledged. First, as a single-center retrospective cohort, the study is subject to potential selection and information bias, and the generalizability of findings requires validation in external populations. Second, Hs-CRP was measured only at baseline without dynamic follow-up, limiting the ability to evaluate longitudinal changes in relation to disease progression. Moreover, due to the retrospective design, several clinically relevant variables—such as ulcer severity (e.g., Wagner grade), bone mineral density, osteoporosis treatment, and exposure to anti-inflammatory agents—were either inconsistently documented or had substantial missing data, preventing their inclusion in the analysis and potentially introducing bias. Third, although multivariable models adjusted for multiple confounders, residual or unmeasured confounding remains possible. Factors such as nutritional status, use of anti-inflammatory medications, and socioeconomic conditions may influence both Hs-CRP levels and mortality risk. For instance, hypoalbuminemia related to poor nutrition may exaggerate the association, while anti-inflammatory drugs could suppress Hs-CRP independently, leading to underestimation. Due to lack of data on variables like treatment adherence, educational level, and comorbidities (e.g., diabetic nephropathy), their potential influence could not be assessed. Fourth, the overall sample size was modest, with especially limited numbers of deaths in certain subgroups (e.g., women, younger patients, non-hypertensive individuals), reducing statistical power and increasing the likelihood of type II errors. These subgroup findings should therefore be interpreted cautiously. Fifth, although two cut-off values for Hs-CRP were applied (median-based and ROC-derived), their clinical utility remains exploratory, as no external validation or head-to-head comparison was conducted. Finally, the study did not evaluate whether interventions targeting Hs-CRP levels could influence outcomes. As no treatment decisions were based on Hs-CRP levels, no causal conclusions can be drawn regarding its role as a modifiable risk factor. To address these limitations and build upon the current findings, future research should focus on prospective multicenter cohort studies to enhance generalizability, incorporate dynamic monitoring of Hs-CRP to assess temporal changes and disease progression, and explore whether targeted interventions to modify Hs-CRP levels can improve clinical outcomes.

## 5 Conclusion

This study first established that Hs-CRP is independently associated with all-cause mortality in patients with osteoporosis and DF. As an inflammatory biomarker and prognostic indicator, Hs-CRP may contribute to improved risk stratification when used alongside conventional clinical factors; however, its role should be interpreted strictly as associative rather than causal due to the study’s retrospective observational design. In addition, key baseline clinical details and follow-up interventions were not fully available due to the retrospective nature of data collection, which further supports cautious interpretation of the findings. The Hs-CRP cut-off values identified in this study (median- and ROC-based thresholds) also require further external validation before they can be applied in routine clinical practice. Future large-scale, multicenter, and preferably prospective studies are needed to confirm these findings, validate clinically applicable cut-off points, and determine whether integrating Hs-CRP with traditional prognostic markers could enhance risk prediction in this high-risk population.

## Data Availability

The raw data supporting the conclusions of this article will be made available by the authors, without undue reservation.
