# Supplementary material for: High-sensitivity C-reactive protein and all-cause mortality in patients with diabetic foot and osteoporosis: evidence from a retrospective cohort study
Source: Front Med (Lausanne). 2026 Jan 12;12:1700752. doi: 10.3389/fmed.2025.1700752 (PMC12832722; doi:10.3389/fmed.2025.1700752)
Supplement: Supplementary file 1 [file Table_1.docx]

| **Table S1.** Test of proportional hazards assumption using Schoenfeld residuals | | |
| --- | --- | --- |
| Variables | Chi-square | P value |
| Age | 0.018 | 0.893 |
| Hypertension | 0.0000975 | 0.992 |
| Lymphocyte count | 3.18 | 0.075 |
| Hemoglobin | 0.00152 | 0.969 |
| FBG | 1.10 | 0.293 |
| HbA1c | 0.00385 | 0.951 |
| HDL-C | 0.325 | 0.569 |
| Uric acid | 1.73 | 0.188 |
| eGFR | 1.13 | 0.288 |
| Albumin | 0.238 | 0.626 |
| Fibrinogen | 0.240 | 0.877 |
| Hs-CRP | 0.692 | 0.405 |
| Global test | 10.4 | 0.581 |

FBG, fasting blood glucose; HbA1c, glycated hemoglobin; HDL-C, high-density lipoprotein cholesterol; eGFR, estimated glomerular filtration rate; Hs-CRP, High-sensitivity C-reactive protein.
